# Early findings from a project aimed at implementing mobile health technology for addiction in primary care

**DOI:** 10.1186/1940-0640-10-S1-A5

**Published:** 2015-02-20

**Authors:** Randall Brown

**Affiliations:** 1 Department of Family Medicine, University of Wisconsin-Madison, Madison, WI, 53706, USA

## Background

More than two-thirds of individuals with a substance use problem have seen a primary care provider in the previous 6 months. However, these substance use disorders often go unrecognized or without treatment due to a lack of optimized assessment and intervention models with which primary care providers are comfortable. As national guidelines call for improved integration of addiction treatment into primary care settings, mobile technology may provide an invaluable tool to primary care providers in assisting their patients in recovery and in improving the efficiency and effectiveness of office visits for these patients. This paper will describe preliminary qualitative data and the early experience with integrating Seva, a suite of mobile technological applications to support recovery, at two Federally Qualified Health Centers (FQHCs).

## Methods

Semistructured interviews were conducted with FQHC patients with substance use disorders at two study sites (Madison, WI: n = 12; Missoula, MT: n = 5) to identify potential strengths and concerns of Seva in addressing substance use problems in primary care. Focus groups were conducted with clinic staff (Madison, WI: n = 20; Missoula, MT: n = 8) to identify strengths and concerns from their perspectives. Notes taken during development meetings between clinic and research teams also provided qualitative data.

## Results

From the clinic perspective, qualitative data indicated the importance of: 1) administrative buy-in; 2) identification of an organizational champion for the intervention; 3) education of clinic staff regarding the research and its importance; 4) achieving a detailed understanding of clinic workflows; 5) the need for transparency regarding the types of information patients enter into Seva that will potentially reach their health-care providers and their medical record; 6) existing gaps in the system that mobile technology might fill for patients in recovery (e.g., providing support for addicted patients living in rural areas, or patients with chaotic lives); and 7) actively addressing concerns of the health-care team regarding the impact of the technology upon workflow. Patient participants expressed concerns regarding confidentiality (primarily from law enforcement and payers) and their desire for access to a support network that is not actively using. Patients viewed Seva features such as recovery meeting times/locations, skills training, and discussion boards (and the network they provide) as strengths.

## Conclusions

Clinicians and patients share the core goals of patient recovery, but they have different needs and different concerns. Implementation will have to address both perspectives. Detailed knowledge of clinic workflows is a key component of implementing patient care innovations on the clinic side, as is minimizing the burden of data collection and monitoring to busy primary care providers. Early dialogue with clinic staff, patients, and administration has been invaluable in achieving understanding of how the technology might facilitate the management of patients in recovery.

## Trial registration

ClinicalTrials.gov NCT01963234

## Grant support

NIDA 5R01DA034279-03

